# Impact of Team-Based Learning on the Clinical Reasoning and Critical Thinking Skills Among Undergraduate Medical Students

**DOI:** 10.7759/cureus.112469

**Published:** 2026-07-11

**Authors:** Debanjana Chowdhury

**Affiliations:** 1 Physiology, Calcutta National Medical College and Hospital, Kolkata, IND

**Keywords:** california critical thinking skill test, clinical reasoning, correlation, critical thinking, critical thinking assessment test, physiology, script concordance test, team-based learning (tbl), undergraduate medical student

## Abstract

Introduction

Critical thinking and clinical reasoning are two core competencies essential for medical students in their evolution from passive learners to proficient future clinicians. These competencies require higher-order cognitive skills which cannot be accomplished through conventional lectures. In contemporary medical education, team-based learning (TBL) has emerged as an active innovative instructional strategy which promotes active participation, accountability, problem-solving, and higher-order thinking. The California Critical Thinking Skills Test (CCTST) assesses critical thinking, while the Script Concordance Test (SCT) measures clinical reasoning under uncertainty. The study aimed to evaluate the effectiveness of TBL in improving critical thinking and clinical reasoning among first-year MBBS students.

Method

An educational interventional study was conducted among 152 first-year MBBS students during a TBL session which included their pre-class preparation, Individual Readiness Assurance Test (IRAT), Team Readiness Assurance Test (TRAT), and application exercises with immediate facilitator feedback. To assess critical thinking, a 20-item questionnaire called the Critical Thinking Assessment Test (CTAT) on adrenal cortex disorders was constructed based on the domains described in the CCTST. To measure clinical reasoning, a 30-item questionnaire was developed on the same topic based on SCT. The two questionnaires which were developed by the investigator and validated by an expert panel were used before and after the TBL session.

Result

A substantial improvement was observed in SCT (Cohen's d=0.89) in comparison to CTAT scores (Cohen's d=0.73) after the TBL session. The TBL intervention strengthened the relationship between clinical reasoning (SCT) and critical thinking (CTAT) transforming a weak correlation (r=0.163; p=0.045) into a moderate, statistically significant correlation (r=0.369; p<0.0001). Student performance increased progressively from IRAT (3.27±1.68) to TRAT (8.97±0.87) to application exercises (11.36±1.35) (p<0.0001).

Conclusion

The study indicates that the implementation of TBL sessions in the early stage of medical training of students can bridge the gap between basic science concepts and clinical applications enhancing both critical thinking and clinical reasoning.

## Introduction

The development of higher-order cognitive skills like clinical reasoning and critical thinking is indispensable in analyzing and interpreting the complexities and uncertainties of patient care, preventing diagnostic error, and helping in therapeutic decision-making to meet the rapidly evolving healthcare requirements [1,2].

Traditional lecture methods often provide limited opportunities to nurture the deeper understanding, logical thinking, and analytical skills of medical students [3,4]. The implementation of active learning strategies like team-based learning (TBL) can be used to foster accountability, team collaboration, problem-solving, and higher-order thinking crucial for a smooth transition from medical students into competent doctors [5-8].

In the 1970s, TBL, first introduced by Dr. Larry Michaelsen in a business school in the United States, is a student-centered instructor-guided educational approach comprising pre-class preparation, the Individual Readiness Assurance Test (IRAT), the Team Readiness Assurance Test (TRAT), application exercise, and immediate feedback and clarification [9,10]. The key advantage of TBL is its ability to provide educational benefits of small group learning in a large classroom setting that requires few facilitators [11].

As evident from the prevalent literature, TBL effectively improves problem-solving, decision-making, and the retrieval of pre-gained knowledge to confront challenges of real-life situations [5-8]. This innovative teaching-learning method helps in enhancing the soft skills like communication, teamwork, and leadership qualities [12,13]. However, there is no study available on the dual effect of critical thinking and clinical reasoning skills when measured concurrently with validated tools such as the California Critical Thinking Skills Test (CCTST) and Script Concordance Test (SCT) [1,2,14]. CCTST is a standardized test to measure the essential components of critical thinking skills: analysis, inference, evaluation, and deductive and inductive reasoning [15-17]. The Critical Thinking Assessment Test (CTAT) based on the five domains of CCTST was designed for this study. SCT is a validated tool devised to assess clinical reasoning under uncertainty [18-20].

The present study aimed to evaluate the impact of TBL in improving critical thinking and clinical reasoning by implementing it on first-year undergraduate medical students in Physiology. This study also intended to analyze the correlation between CTAT and SCT scores as well as to assess the perception and satisfaction of the students with the TBL session.

The correlation between CTAT and SCT can provide insights into how critical thinking skills are associated with clinical reasoning which ultimately will prepare the students to provide safe and effective patient care.

## Materials and methods

An educational interventional study was carried out in the Department of Physiology of Calcutta National Medical College and Hospital in Kolkata, India, among first-year undergraduate medical students of the academic session 2024-2025. The study was conducted from April 2025 to September 2025 after obtaining necessary approval from the Institutional Ethics Committee of Calcutta National Medical College (approval number: EC-CNMC/600/2025) dated 08/02/2025 and informed consent of the participants.

An orientation session of one hour duration was held on the first day to explain the study procedure to the participants. As the physiology of adrenal gland hormones had already been covered through lecture classes, disorders of the adrenal cortex like Cushing's syndrome, Conn's syndrome, and Addison's disease were selected as the topic for the TBL session. On the second day, two pre-tests, one for assessing critical thinking and another for clinical reasoning skills, were performed using two separate questionnaires before the actual TBL session. A 20-item questionnaire called CTAT consisting of questions on adrenal cortex disorders based on the conceptual framework of CCTST was used to assess the critical thinking skills of the students [15,16].

The investigator developed CTAT inspired by CCTST which focuses on five domains (Appendix A). Firstly, the Analysis domain involves identifying problems, recognizing relationships, and interpreting information. Secondly, the Evaluation domain judges the credibility of statements and the strength of arguments. Thirdly, the Inference domain draws logical conclusions from the data. The next domain, called Deductive Reasoning, uses general rules to reach specific, certain, and guaranteed conclusions. Lastly, the Inductive Reasoning domain identifies patterns to determine the most probable but not guaranteed conclusion [15,16].

Evaluation of clinical reasoning under uncertainty was accomplished utilizing a relatively novel assessment tool called SCT (Appendix B). For this, a 30-item SCT was constructed by using realistic practical situations called clinical vignettes on adrenal disorders [18-20]. Each vignette was followed by a diagnostic or management hypothesis and a new clinical finding. Students were instructed to judge how the new information influenced the proposed hypothesis using a 5-point Likert scale of -2 to +2 where +2 means strongly supports the hypothesis; +1, slightly supports the hypothesis; 0, no effect; -1, slightly weakens the hypothesis; and -2, strongly weakens the hypothesis.

For instance, a 30-year-old woman presents with recent weight gain, a rounded face, and purple striae. She has hypertension and diabetes. She reported irregular periods, pain, and proximal muscle weakness. The effect of new information on the hypothesis is illustrated in Table 1.

**Table 1:** New information on hypothesis PCOS: polycystic ovary syndrome; ACTH: adrenocorticotropic hormone

Hypothesis	New information	Answer
1. She is suffering from Cushing syndrome and not PCOS.	Serum cortisol level is elevated at midnight.	-1, -2, 0, +1, +2
2. She has Cushing disease.	Her cortisol level is not suppressed with low-dose dexamethasone but is suppressed with high-dose dexamethasone.	-1, -2, 0, +1, +2
3. She has adrenal adenoma.	ACTH is already suppressed.	-1, -2, 0, +1, +2
4. Her hypertension is due to essential hypertension.	Renin activity is suppressed.	-1, -2, 0, +1, +2
5. Muscle weakness is due to hypokalemia.	Serum K+=2.8 mmol/L.	-1, -2, 0, +1, +2

A reference panel of seven content experts, five from Physiology, one from General Medicine, and one from Endocrinology, independently answered all SCT items according to their professional judgment. The reference answer for each item was the response opted for by the highest number of experts (modal response). Partial credit (X) scoring was derived with the help of an aggregate scoring method using the following formula: $\mathrm{X}=\frac{\text{Number of experts selecting the response}}{\text{Number of experts selecting the modal response}}$. 

An example SCT scoring (aggregate scoring method) is shown in Table 2.

**Table 2:** Example SCT scoring (aggregate scoring method) SCT: Script Concordance Test

Response options	-2	-1	0	1	2	Calculation of credits
Number of experts selecting response for Item 1	0	0	1	1	5	Total experts = 7
Credit calculation	0/5	0/5	1/5	1/5	5/5	Modal response = +2
Final scores	0	0	0.20	0.20	1.00	Rounded to 2 decimals

The content validity of two self-developed assessment tools regarding relevance and clarity was established by a panel of seven experts by reviewing each item meticulously. The Item-Level Content Validity Index (I-CVI) and Scale-Level Content Validity Index (S-CVI) were calculated on the basis of expert ratings. The I-CVI varied from 0.86 to 1.00 in the case of both tools. The S-CVI/average was 0.93 for CTAT and 0.96 for SCT suggesting excellent content validity.

Out of 250 students, only 152 participants who volunteered and attended all the steps of the TBL session were randomly divided into four groups, A, B, C, and D, with 42, 40, 36, and 34 students, respectively. The students were provided with the study material on adrenal cortex disorders for preparation outside the classroom. After one week, a two-hour TBL session was initiated with IRAT consisting of 10 multiple-choice questions (MCQ) to assess the preparedness of the individual students (Appendix C). Each group was further subdivided into small teams comprising 6-7 members. Then, students of each team collaborated and were engaged in peer discussion to answer TRAT having the same MCQ as IRAT using cards. After that, the facilitator discussed the correct answers with explanations, clarified misconceptions, and provided immediate feedback. This was followed by three application exercises on adrenal gland disorders designed in conformity with the 4 "S" of the TBL exercise: Significant problem, Same problem, Specific choice, and Simultaneous reporting [9] (Appendix D). The team members were instructed to discuss and analyze the problem by integrating physiological concepts and find the solution by applying their prior knowledge. The responses to these problem-solving exercises are scored out of 15 marks. On the final day, post-tests on the same CTAT and SCT were administered to evaluate the improvement after the TBL session. A 10-item feedback questionnaire based on a 5-point Likert scale was distributed to assess their acceptance, satisfaction, and perception regarding the implementation of the TBL session (Appendix E). Satisfaction index (SI) was calculated using the following formula: $SI = \frac{\sum (f \times s)}{N \times S} \times 100$. Here, f is the frequency of response, s is the score of the response option, N is the total number of respondents, and S is the highest possible score (for a 5-point scale=5).

**Figure 1:**
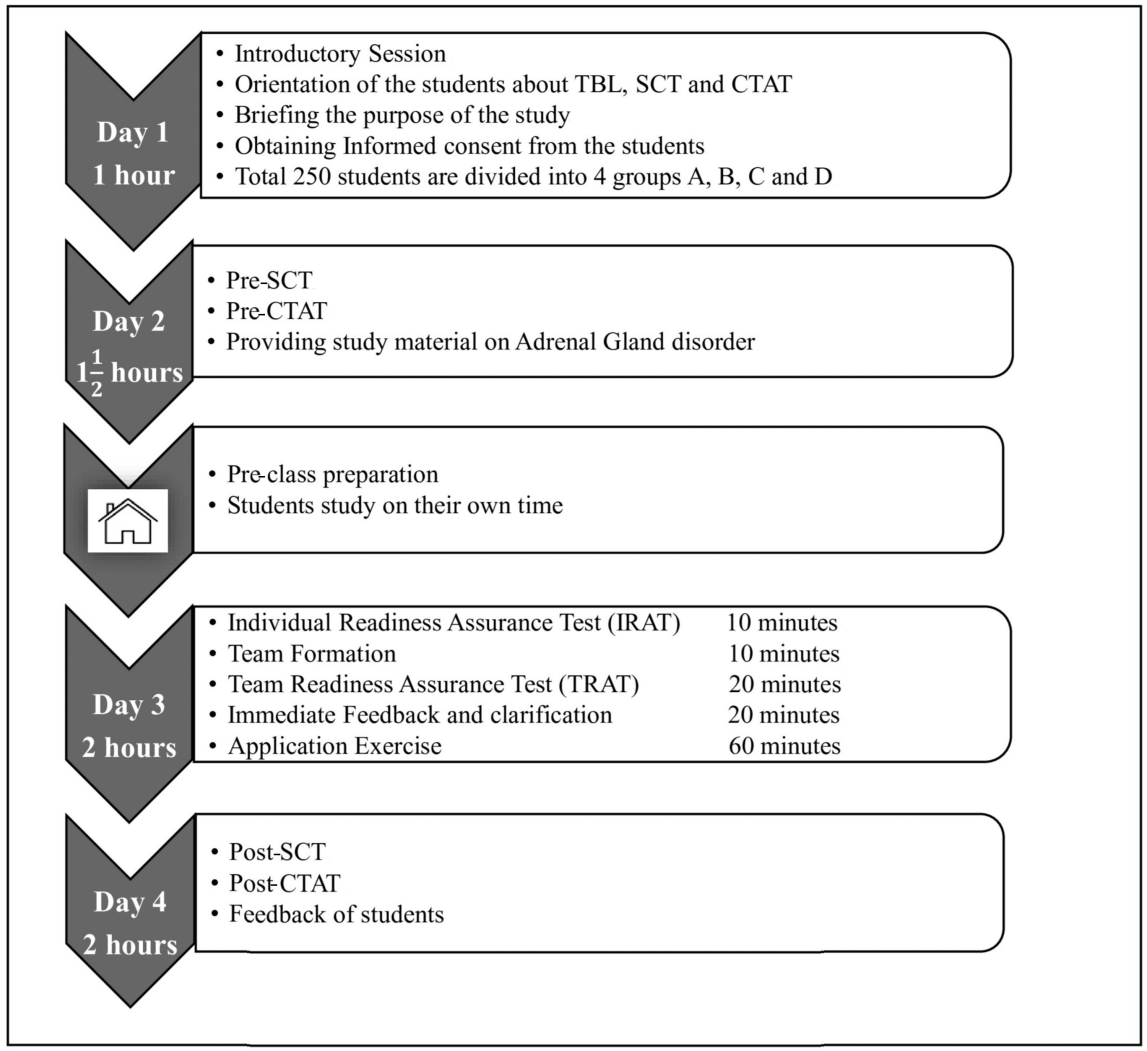
Structure of TBL on adrenal disorder in Physiology for first-year MBBS students TBL: team-based learning; CTAT: Critical Thinking Assessment Test; SCT: Script Concordance Test This figure was created using Microsoft Word and PowerPoint (Microsoft Corporation, Redmond, Washington, United States).

 The steps of the TBL session are elaborated in Figure 1.

A pilot study was conducted on 30 students of the 2023-2024 batch using the newly developed SCT, CTAT, and feedback questionnaire to test the validity. Cronbach's alpha for SCT, CTAT, and feedback were found to be 0.88, 0.87, and 0.96, respectively.

Statistical analysis

Data were analyzed using IBM SPSS Statistics for Windows, Version 25.0 (IBM Corp., Armonk, New York, United States). Pre- and post-test SCT and CTAT scores were compared using the paired t-test, with the effect size having been calculated by Cohen's d. IRAT, TRAT, and application exercise scores were compared using one-way ANOVA. Correlation between SCT and CTAT scores was assessed using Pearson's correlation coefficient analysis. Feedback was analyzed using SI. A p-value of <0.05 was considered to be statistically significant.

## Results

Out of 250 Phase I MBBS students, 152 attended TBL sessions on adrenal disorders. The participants (age: 18.67±0.91 years) consisted of 64 (42.11%) female and 88 (57.89%) male students. For TBL implementation, four groups, A, B, C, and D, consisting of 42, 40, 36, and 34 students, respectively, were formed.

Table 3 shows a statistically significant improvement in SCT scores following the TBL session across all groups (p<0.0001). The overall mean pre-test score increased from 9.90±3.77 to a post-test score of 17.85±7.11, a gain of 7.95 with a large effect size (Cohen's d=0.89) denoting substantial educational impact.

**Table 3:** Comparison of the pre-test and post-test scores of students of the Script Concordance Test before and after the team-based learning session A total of 152 students out of 250 participated in the study. The participants were divided into four groups: A, B, C, and D. The p-value was obtained using paired t-test.

Groups	Pre-test	Post-test	Cohen's d	Gain	P-value
	Mean±SD	Mean±SD			
Group A (n=42)	9.68±3.99	18.26±7.51	0.89	8.58	<0.0001
Group B (n=40)	9.51±4.31	18.22±7.30	0.89	8.71	<0.0001
Group C (n=36)	10.42±3.46	17.58±6.77	0.87	7.16	<0.0001
Group D (n=34)	10.10±3.16	17.24±6.98	0.89	7.14	<0.0001
Total students (n=152)	9.90±3.77	17.85±7.11	0.89	7.95	<0.0001

Table 4 depicts a statistically significant improvement in CTAT scores (p<0.0001). The overall mean score improved from 8.74±2.99 to 13.03±4.56, showing a gain of 4.29 with moderate effect sizes (Cohen's d=0.73).

**Table 4:** Comparison of the pre-test and post-test scores of students of the Critical Thinking Assessment Test before and after the team-based learning session A total of 152 students out of 250 participated in the study. The participants were divided into four groups: A, B, C, and D. The p-value was obtained using paired t-test.

Groups	Pre-test	Post-test	Cohen's d	Gain	P-value
	Mean±SD	Mean±SD			
Group A (n=42)	8.83±3.19	13.38±4.46	0.71	4.55	<0.0001
Group B (n=40)	8.45±3.03	13.27±5.08	0.76	4.82	<0.0001
Group C (n=36)	8.92±2.98	12.78±4.15	0.72	3.86	<0.0001
Group D (n=34)	8.79±2.78	12.56±4.57	0.72	3.77	<0.0001
Total students (n=152)	8.74±2.99	13.03±4.56	0.73	4.29	<0.0001

**Figure 2:**
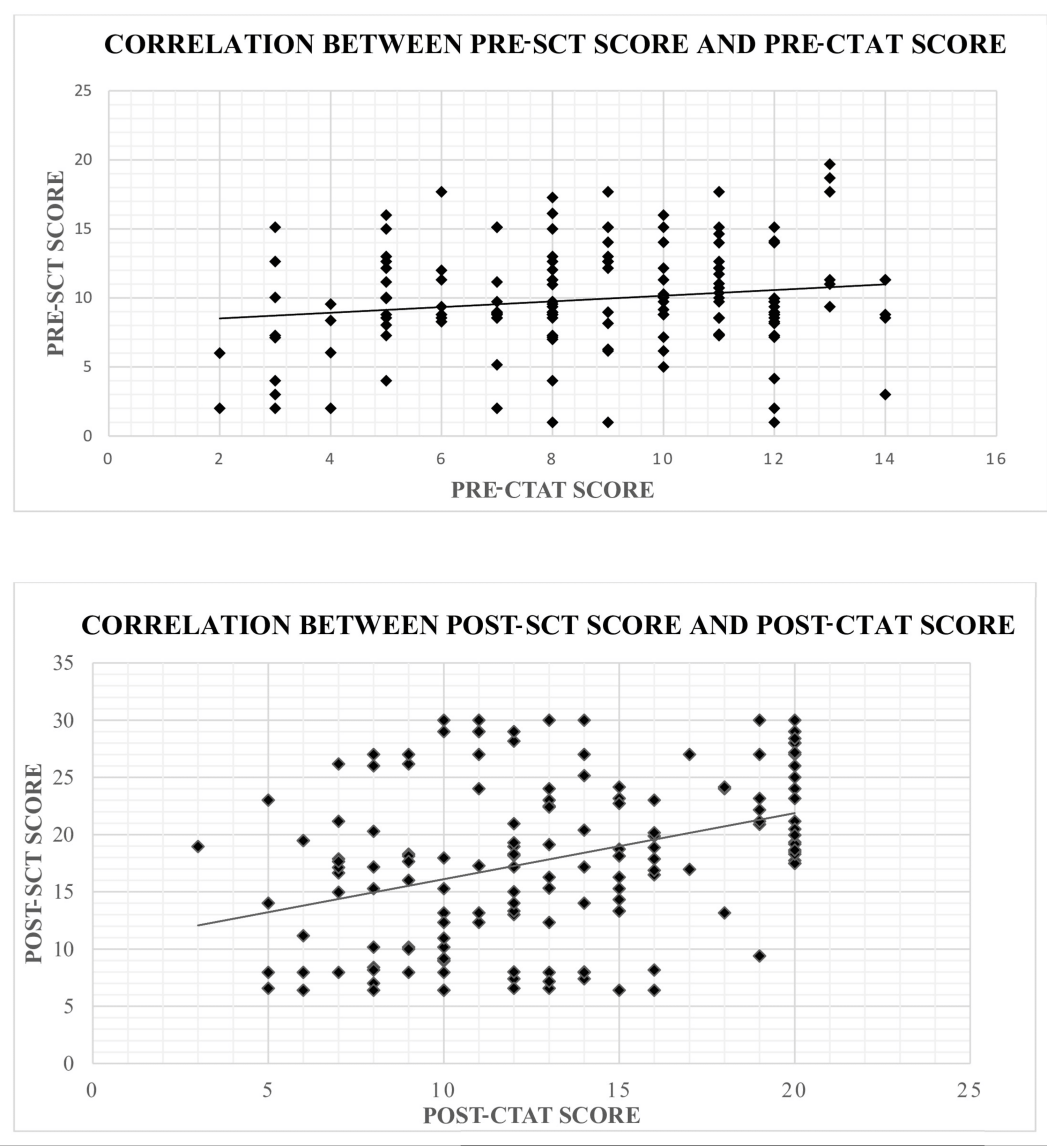
Pearson's correlation between CTAT and SCT scores before and after the team-based learning Correlation between the pre-test scores of CTAT and SCT was weak (r=0.163; p=0.045) but increased to a moderate and positive correlation (r=0.369; p<0.0001) after the team-based learning implementation. CTAT: Critical Thinking Assessment Test; SCT: Script Concordance Test

Figure 2 represents Pearson's correlation between the pre-test scores of CTAT and SCT, which was weak (r=0.163; p=0.045) but increased to a moderate positive correlation (r=0.369; p<0.0001) after TBL implementation.

Table 5 displays a significant progressive improvement in scores from IRAT (3.27±1.68) to TRAT (8.97±0.87) and further to application exercises (11.36±1.35) across all groups, reflecting progressive enhanced performance through the TBL structure (p<0.0001).

**Table 5:** Comparison of IRAT, TRAT and application exercise scores of students after the team-based learning session A total of 152 students out of 250 participated in the study. The participants were divided into four groups: A, B, C, and D. The p-value was obtained using one-way ANOVA. IRAT: Individual Readiness Assurance Test; TRAT: Team Readiness Assurance Test

Groups	IRAT	TRAT	Application exercise	P-value
	Mean±SD	Mean±SD	Mean±SD	
Group A (n=42)	3.07±1.72	9.17±0.69	11.52±0.96	<0.0001
Group B (n=40)	3.35±1.67	9.20±0.76	11.60±1.37	<0.0001
Group C (n=36)	3.42±1.77	8.83±1.08	11.17±1.59	<0.0001
Group D (n=34)	3.26±1.58	8.62±0.82	11.06±1.41	<0.0001
Total students (n=152)	3.27±1.68	8.97±0.87	11.36±1.35	<0.0001

As evident from Table 6, students expressed a high level of satisfaction with TBL (SI: 78.42-87.50), with the majority agreeing about strengthening their critical thinking, clinical reasoning, teamwork, integration of knowledge, and promotion of deeper conceptual understanding.

**Table 6:** Feedback of students on TBL on adrenal disorders using Likert scale A total of 152 students out of 250 participated in the study. IRAT: Individual Readiness Assurance Test; TRAT: Team Readiness Assurance Test; TBL: team-based learning; CTAT: Critical Thinking Assessment Test; SCT: Script Concordance Test

Questions	Strongly agree (5)	Agree (4)	Neutral (3)	Disagree (2)	Strongly disagree (1)	Satisfaction index
	n	n	n	n	n	(%)
1. The IRAT was effective in assessing my preparation.	40	78	21	8	5	78.42
2. The TRAT enhanced my understanding of the concepts.	56	62	18	9	7	79.87
3. Working in teams improved my participation, collaboration, and willingness to share ideas.	58	60	22	7	5	80.92
4. The TBL session encouraged me to analyze clinical information rather than just memorizing facts.	61	64	17	6	4	82.63
5. The questions of CTAT were challenging to understand and answer.	64	67	15	4	2	84.61
6. The TBL session improved my critical thinking skills.	78	58	12	3	1	87.5
7. The clinical cases helped me to apply physiological concepts to real-world situations.	65	62	16	6	3	83.68
8. The SCT enhanced my clinical reasoning and decision-making ability.	72	61	15	3	1	86.32
9. The TBL session helped me to integrate knowledge from multiple domains while solving the cases.	74	54	14	6	4	84.74
10. I prefer the TBL session over didactic lecture for in-depth understanding of the subject and improvement of interpersonal skills.	76	52	17	5	2	85.66

Cronbach's alpha of the feedback questionnaire, SCT, and CTAT were 0.95, 0.89, and 0.84, respectively, which demonstrated good internal consistency and reliability.

## Discussion

The present study elucidates that TBL is an effective educational approach for enhancing clinical reasoning and critical thinking among first-year MBBS students. The greater improvement in SCT scores (Cohen's d=0.89) compared to CTAT (Cohen's d=0.73) is attributed to the fact that structured scenario-based TBL closely aligns with the SCT which assesses the reasoning ability under uncertainty and interprets data to make a decision. On the contrary, the CTAT assesses complex critical thinking skills that require repeated reinforcement over a longer period, being less responsive to short context-specific instructional intervention.

A key finding of this study was the shift from weak correlation (r=0.163; p=0.045) between SCT and CTAT scores before TBL session to a moderate positive correlation (r=0.369; p<0.0001) after TBL. This improvement is due to the structural framework of TBL, particularly team application exercises, where students share logical perspectives, engage in peer discussion, reflect on misconceptions, and refine their opinions with expert feedback enabling them to effectively translate their analytical skills while navigating complex uncertain clinical situations and securing patient safety [11,14].

Clinical reasoning is a fundamental competency in medical education encompassing the integration and interpretation of patient information to develop differential diagnosis and decide correct treatment to ensure comprehensive patient care [1,10,18]. Critical thinking is a high-level rational thinking process that helps to analyze and evaluate information logically to arrive at the best possible solution to the clinical problem [2,16,17]. SCT and CTAT have been included in this study since standard assessments like MCQ cannot assess the complex reasoning, clinical judgement, and critical thinking skills essential for clinical practice.

Existing literature reveals that TBL is an innovative transferable pedagogical strategy that fosters problem-solving, critical thinking, clinical reasoning, decision-making, team collaboration, and higher-order cognitive skills [5-8,11-14]. However, the relationship between clinical reasoning and critical thinking after TBL sessions in medical students has not been documented in any prior study. The present findings highlight that well-organized interactive learning can connect theoretical knowledge and clinical application leading to the comprehensive understanding and integration of concepts.

The score progressed from IRAT to TRAT and subsequently to application exercise due to individual accountability, peer learning, student engagement, and collaborative problem-solving features of TBL. The observed trends are consistent with existing literature indicating the positive influence of TBL on learning outcomes [5-8].

Students also preferred TBL over traditional didactic lectures which corroborates with previous studies [3-8]. TBL is an efficacious educational strategy for promoting critical thinking and clinical reasoning in a large group of medical students which require limited faculty resources.

Limitations

The study was conducted in one particular institution on a limited number of students over a short period of time which may restrict the generalization of the findings. Another shortcoming of the present study is that the qualitative analysis of the student feedback could not be incorporated which could further enhance the depth of the study.

## Conclusions

This study emphasizes the importance of incorporating TBL with assessment tools like SCT and CTAT in the medical curriculum to enhance the clinical reasoning and critical thinking necessary for future expert medical practitioners. These methods will help the student to transcend from rote learning and factual recall to higher-order thinking skills for professional development. Future research could explore whether repeated exposure to TBL sessions across diverse topics of Physiology, involving more students, may lead to greater cognitive development necessary for critical thinking and clinical reasoning skills, thereby promoting the long-term retention of knowledge.

## Appendices

Appendix A

Critical Thinking Assessment Test (CTAT) Questions Based on the Five Domains of the California Critical Thinking Skills Test (CCTST)

There are four questions in each domain, totaling 20 questions. Total marks=1×20=20 marks

I. Analysis

1. A 25-year-old woman presents with chronic fatigue, dizziness on standing, nausea, and skin pigmentation. Her lab results show the following: serum sodium: 124 mmol/dL; serum potassium: 5.9 mmol/dL; blood glucose: 60 mg/dL; serum cortisol: low; and ACTH: high. Two students propose explanations for the hypotension: Student M: The hypotension is due to aldosterone deficiency leading to sodium and water loss. Student N: The hypotension is due to cortisol deficiency impairing vascular responsiveness to catecholamines. Which student's reasoning is better supported by the evidence?

(a) Only Student M is correct. (b) Only Student N is correct. (c) Both Student M and Student N are correct. (d) Neither Student M nor Student N is correct.

2. A 40-year-old woman presents with central obesity, facial rounding, and purple striae on her abdomen. She reports easy bruising and muscle weakness. Laboratory results show the following: 24-hour urinary free cortisol: elevated; midnight salivary cortisol: elevated; morning ACTH: low; and dexamethasone suppression test: cortisol not suppressed. An MRI of the pituitary is normal. Which of the following statements best analyzes the relationship between these findings and the likely cause of hypercortisolism?

(a) The elevated urinary cortisol confirms Cushing's syndrome, but low ACTH suggests an adrenal source rather than pituitary. (b) The normal pituitary MRI rules out Cushing's disease; therefore, it must be iatrogenic. (c) Purple striae are the most specific finding confirming Cushing's disease. (d) The dexamethasone suppression test is unreliable and should be ignored in analysis.

3. A 58-year-old male with treatment-resistant hypertension presents with fatigue and muscle cramps. His lab results show hypokalemia, hypernatremia, and a high aldosterone-to-renin ratio. Further investigation with an adrenal CT scan reveals a 2.5 cm solitary adenoma on the left adrenal gland. Based on this presentation, what is the most logical next step in diagnosis and treatment?

(a) Prescribe spironolactone to manage the symptoms and monitor blood pressure. (b) Proceed directly to laparoscopic adrenalectomy to remove the adenoma. (c) Perform adrenal venous sampling to confirm the unilateral source of aldosterone excess. (d) Begin a low-sodium diet and re-evaluate the patient's aldosterone-to-renin ratio in six weeks.

4. A 25-year-old woman presents with fatigue, weight loss, and hyperpigmentation of her skin and oral mucosa. Her blood pressure is 90/60 mm Hg. Laboratory investigations show the following: serum sodium: 128 mEq/L; serum potassium: 5.8 mEq/L; morning serum cortisol: 3 µg/dL (low); and plasma ACTH: markedly elevated. She reports that her symptoms worsen during periods of stress and that she recently stopped long-term steroid therapy. Which of the following best analyzes the relationship between the lab findings and the possible cause of her condition?

(a) The elevated ACTH and low cortisol indicate adrenal failure, consistent with primary Addison's disease. (b) The low sodium and high potassium indicate kidney failure rather than adrenal dysfunction. (c) The elevated ACTH suggests secondary (pituitary) adrenal insufficiency. (d) The low cortisol is due to the sudden withdrawal of steroids, confirming Cushing's syndrome.

II. Inference

1. A 55-year-old man presents with rapidly progressive muscle weakness, severe hypertension, newly onset diabetes, hypernatremia, hypokalemia, and metabolic alkalosis. Laboratory tests show the following: serum cortisol: high; ACTH: increased; and cortisol: not suppressed with high-dose dexamethasone. What is the likely diagnosis?

(a) Small cell carcinoma of the lung. (b) Pituitary adenoma. (c) Adrenal adenoma. (d) Hypothalamic tumour.

2. A 45-year-old patient with long-standing hypertension is found to have low serum potassium and metabolic alkalosis. Imaging reveals a unilateral adrenal adenoma. Her physician, suspecting Conn's syndrome, correctly orders an aldosterone-to-renin ratio. Given the patient's findings, which of the following is the most likely outcome of the aldosterone-to-renin ratio test?

(a) Both aldosterone and renin levels will be abnormally low. (b) Aldosterone will be high, and renin will be low. (c) Aldosterone will be low, and renin will be high. (d) Both aldosterone and renin levels will be abnormally high.

3. A 25-year-old male with a history of an autoimmune thyroid disorder presents with chronic fatigue, weight loss, and darkening of his skin, particularly on his knuckles and oral mucosa. Lab tests show low cortisol and low blood sodium. Based on the patient's autoimmune history and clinical presentation, the most logical interference is that the hyperpigmentation is a direct consequence of:

(a) Increased production of melanin due to elevated ACTH. (b) An infection-induced process affecting the skin cells. (c) Decreased peripheral blood flow causing skin changes. (d) A separate autoimmune attack on the melanocytes.

4. A 30-year-old woman presents with fatigue, loss of appetite, and unintentional weight loss for 3 months. She reports darkening of the skin, particularly over her elbows and gums. Her laboratory findings are as follows: blood pressure: 88/60 mmHg; serum sodium: 126 mEq/L; serum potassium: 5.6 mEq/L; morning cortisol: 2.5 µg/dL (low); and plasma ACTH: 110 pg/mL (elevated). From the information provided, which of the following can be most reasonably inferred about the underlying disorder?

(a) The adrenal cortex is failing to respond to ACTH, leading to cortisol deficiency. (b) The pituitary gland is under-secreting ACTH, causing low cortisol levels. (c) The symptoms are caused by excessive cortisol production from an adrenal tumour. (d) The electrolyte imbalance is unrelated to adrenal function.

III. Evaluation

1. A student claims "Hypertension in Cushing's syndrome is due to aldosterone". Which statement best evaluates this claim?

(a) Correct, increased aldosterone is the main cause of Cushing's syndrome. (b) Incorrect, Cushing's is associated with cortisol-induced mineralocorticoid effects. (c) Correct, cortisol increases aldosterone production. (d) Incorrect, cortisol suppresses aldosterone secretion.

2. Claim: "All patients with hypertension and low potassium should be suspected for Conn's syndrome".

(a) Accept it because the findings are diagnostic. (b) Accept it because hypokalemia is uncommon in hypertension. (c) Reject it because hypokalemia may be due to diuretics. (d) Reject it because aldosterone does not affect potassium.

3. A 30-year-old female presents with recent weight gain, easy bruising, and irregular menstrual cycles. Her physician orders a 24-hour urine free cortisol (UFC) test, which is elevated. The physician confidently tells the patient that she has Cushing's syndrome and that a high-dose dexamethasone suppression test (HDDST) is not necessary. Evaluate the physician's diagnostic approach. Which of the following is the most accurate assessment of the physician's reasoning?

(a) The physician's approach is sound, as a high UFC is sufficient for diagnosing Cushing's syndrome. (b) The physician is overconfident; while the UFC suggests Cushing's syndrome, it is not specific enough to determine the underlying cause without further testing. (c) The physician is correct in skipping the HDDST, as imaging alone can differentiate between the causes of hypercortisolism. (d) The physician is incorrect; the HDDST is the only test needed to diagnose and localize Cushing's syndrome.

4. A 40-year-old patient presents with symptoms suggestive of Addison's disease: fatigue, weight loss, and hyperpigmentation. A baseline cortisol level is found to be low. The physician performs an ACTH stimulation test. The cortisol level does not rise appropriately after ACTH administration, confirming the diagnosis of primary adrenal insufficiency. The physician, however, argues that testing for autoimmune antibodies is unnecessary because the diagnosis is already confirmed. Evaluate the physician's argument. Why is this reasoning incomplete or potentially flawed?

(a) Autoimmune testing is never necessary in Addison's disease as the diagnosis is purely clinical. (b) While the diagnosis is confirmed, establishing the underlying cause (e.g., autoimmune vs. infectious) is crucial for long-term management and screening for other associated conditions. (c) The ACTH stimulation test is unreliable, so autoimmune testing is the only way to accurately diagnose Addison's disease. (d) Autoimmune testing is only required if the ACTH stimulation test was equivocal.

IV. Deduction

1. A patient is confirmed to have a solitary adrenal adenoma causing Conn's syndrome. Medical management with spironolactone is initiated. Based on the general principle, what can be logically deduced to be the mechanism by which spironolactone will improve the patient's symptoms?

(a) It inhibits the production of aldosterone by the adrenal adenoma. (b) It directly blocks the reabsorption of sodium in the kidneys. (c) It antagonizes the effect of aldosterone at the mineralocorticoid receptor in the kidney. (d) It increases the level of renin, thereby balancing the aldosterone excess. 

2. A 35-year-old male with a newly diagnosed primary adrenal insufficiency presents to the emergency department in an "adrenal crisis" with severe hypotension and confusion. Based on the general principle, what is the most critically and immediately required therapeutic intervention to address the patient's severe hypotension?

(a) Administration of a high dose of intravenous (IV) corticosteroids. (b) Administration of a mineralocorticoid, such as fludrocortisone. (c) Intravenous fluid resuscitation with saline to correct volume depletion. (d) A high dose of oral corticosteroids with a mineralocorticoid. 

3. What would be the plasma glucose level in untreated Addison's disease likely show?

(a) Normal. (b) Increased. (c) Decreased. (d) Variable.

4. A 35-year-old woman presents with progressive weight gain over the abdomen and face, easy bruising, and menstrual irregularities. Investigations reveal the following: 24-hour urinary free cortisol: ↑ markedly elevated; plasma ACTH: ↑ elevated; low-dose dexamethasone suppression test: no suppression; high-dose dexamethasone suppression test: cortisol levels decrease significantly; and MRI of the pituitary: small adenoma identified. Based on the normal physiological feedback control of the HPA axis and these test results, which of the following must be true?

(a) The cortisol excess originates from an adrenal adenoma that is independent of ACTH control. (b) The source of excess ACTH is the pituitary, because high-dose dexamethasone partially suppresses cortisol production. (c) The elevated ACTH is due to ectopic secretion from a non-pituitary tumour that is unresponsive to dexamethasone. (d) The cortisol excess is due to chronic stress-induced hypersecretion from the hypothalamus.

V. Induction

1. A 35-year-old male has been diagnosed with Cushing's syndrome. Initial testing shows high cortisol but low ACTH levels. His physician is deciding on the next diagnostic step. What is the most logical next step in the diagnostic process?

(a) A pituitary MRI. (b) An inferior petrosal sinus sampling test (IPSS) test. (c) Imaging (CT scan) of the adrenal glands. (d) A high-dose dexamethasone suppression test.

2. A 60-year-old patient has a history of high blood pressure that has been difficult to control with standard anti-hypertensive medications. Recent lab results consistently show low serum potassium (hypokalemia) despite no diuretic use. An aldosterone-to-renin ratio is found to be significantly elevated. Which of the following generalizations can be most reliably induced from these specific findings?

(a) All patients with hypokalemia must have Conn's syndrome. (b) In resistant hypertension, the underlying cause is likely an adrenal gland issue. (c) Primary hyperaldosteronism, often due to an adenoma, causes high blood pressure and persistent hypokalemia by a specific hormonal imbalance. (d) A high aldosterone-to-renin ratio is a conclusive finding for any patient with high blood pressure.

3. A series of patients present with fatigue and hypotension. Their lab findings are summarized in Table 7.

**Table 7:** Details of the three patients ACTH: adrenocorticotropic hormone

Patient	Na⁺ (mEq/L)	K⁺ (mEq/L)	Cortisol (µg/dL)	ACTH (pg/mL)	Adrenal CT
						A	126	5.8	2	120	Normal
B	138	4	12	35	Normal
C	125	6	3.5	140	Normal

From the observed pattern, which general inference is most reasonable?

(a) Low cortisol with high ACTH and normal imaging suggests autoimmune adrenal destruction. (b) Low cortisol with low ACTH indicates secondary pituitary failure. (c) Normal imaging and normal hormones exclude adrenal disease. (d) High ACTH and high cortisol suggest Cushing's disease.

4. A clinician reviews 15 cases of secondary hypertension. Patterns observed were as follows: patients with low renin and high aldosterone → primary hyperaldosteronism (Conn's); patients with high renin and high aldosterone → renal artery stenosis; and patients with normal renin and aldosterone → essential hypertension. A new hypertensive patient has the following: serum sodium: 150 mEq/L; serum potassium: 2.9 mEq/L; plasma renin: 0.2 ng/mL/hr (suppressed); plasma aldosterone: 35 ng/dL (elevated); and adrenal CT: left adrenal nodule. Based on the recurring relationships seen in prior patients, what is the most reasonable inference about this new case?

(a) It most likely represents primary hyperaldosteronism due to an adrenal adenoma. (b) The hypertension is due to renal artery narrowing and secondary aldosteronism. (c) The hormonal changes are nonspecific and unrelated to the adrenal gland. (d) The data are insufficient to make any probable conclusion.

Appendix B

Script Concordance Test (SCT) to Assess Clinical Reasoning Before and After a Team-Based Learning (TBL) Session on Adrenal Disorders in First-Year Medical Students

There are six vignettes, with 5 questions in each vignette, totaling 30 questions. Total marks=1×30=30 marks

Instructions: For each item, judge the impact of the new information on the proposed hypothesis.

Use the following scale: +2 = Strongly supports the hypothesis +1 = Slightly supports the hypothesis 0 = No effect -1 = Slightly weakens the hypothesis -2 = Strongly weakens the hypothesis

Vignette 1

A 30-year-old woman presents with recent weight gain, a rounded face, and purple striae. She has hypertension and diabetes. She reported irregular periods, ache, and proximal muscle weakness. See Table 8 and answer.

**Table 8:** Vignette 1 PCOS: polycystic ovary syndrome; ACTH: adrenocorticotropic hormone

Hypothesis	New information	Answer
1. She is suffering from Cushing syndrome and not PCOS.	Serum cortisol level is elevated at midnight.	
2. She has Cushing disease.	Her cortisol level is not suppressed with low-dose dexamethasone but is suppressed with high-dose dexamethasone.	
3. She has adrenal adenoma.	ACTH is already suppressed.	
4. Her hypertension is due to essential hypertension.	Renin activity is suppressed.	
5. Muscle weakness is due to hypokalemia.	Serum K+=2.8 mmol/L.	

Vignette 2

A 30-year-old woman presents with central obesity, a rounded face, and purple striae. She has hypertension and diabetes. She reported irregular periods, ache, and proximal muscle weakness. See Table 9 and answer.

**Table 9:** Vignette 2 DEXA: dual-energy X-ray absorptiometry; ACTH: adrenocorticotropic hormone; RA: rheumatoid arthritis

Hypothesis	New information	Answer
1. Her osteoporosis is due to Cushing syndrome.	DEXA scan shows low mineral density with vertebral fractures.	
2. Her metabolic alkalosis is due to potassium depletion.	Arterial blood gas shows the following: pH=7.52 and HCO3-=34 mmol/L.	
3. She has iatrogenic Cushing's syndrome.	She reports long-term oral prednisolone use for RA.	
4. Surgical resection/transsphenoidal surgery is the best treatment.	MRI shows a well-localized 5 mm pituitary microadenoma. ACTH is elevated.	
5. Medical therapy with ketoconazole would be effective.	Lab shows increased cortisol with inoperable adrenal carcinoma.	

Vignette 3

A 40-year-old male presents with fatigue, weakness, weight loss, abdominal pain, nausea, and vomiting. See Table 10 and answer.

**Table 10:** Vignette 3 ACTH: adrenocorticotropic hormone

Hypothesis	New information	Answer
1. He has primary adrenal insufficiency.	Serum sodium=126 mmol/L. Serum potassium=5.8 mmol/L. Hyperpigmentation over the skin, buccal mucosa, and palmar creases.	
2. He is suffering from primary adrenal insufficiency (Addison's disease).	Serum cortisol level is low. ACTH is high.	
3. The diagnosis of Addison's disease is correct.	Plasma renin is high.	
4. His diagnosis is confirmed.	ACTH stimulation test shows a cortisol rise of >18 ug/dL after 30 minutes.	
5. He has secondary adrenal insufficiency.	Plasma ACTH is low. Absence of hyperpigmentation.	

Vignette 4

A 40-year-old male presents with fatigue, weakness, weight loss, abdominal pain, nausea, and vomiting. He was on long-term steroid therapy. See Table 11 and answer.

**Table 11:** Vignette 4

Hypothesis	New information	Answer
1. He is in an Addisonian crisis.	Serum sodium=140 mmol/L. Serum potassium=6 mmol/L. Blood glucose=60 mg/dL. Blood pressure=90/60 mmHg. He skipped his steroid medication for the last 7 days.	
2. The steroid dose should be increased.	He developed high-grade fever with lower respiratory tract infections.	
3. Treatment with IV fluids only is sufficient.	Central venous pressure is low. Severe hypotension. Heart rate 120/min. Confusion, dizziness, and altered mental status noted.	
4. IV hydrocortisone therapy is essential.	Patient is in shock despite adequate IV fluids.	
5. Mineralocorticoid/fludrocortisone should be added.	Despite adequate hydrocortisone, she has hypotension, hyponatremia, and hyperkalemia.	

Vignette 5

A 38-year-old man presents with persistent hypertension, hypokalemia, and metabolic alkalosis. See Table 12 and answer.

**Table 12:** Vignette 5

Hypothesis	New information	Answer
1. He is suffering from primary hyperaldosteronism.	Edema absent. Aldosterone increased. Renin decreased. Fludrocortisone suppression test: aldosterone secretion not suppressed.	
2. He has Conn syndrome.	Plasma aldosterone-to-renin ratio <10.	
3. The cause of these symptoms is adrenal hyperplasia.	Adrenal vein sampling confirms unilateral excess.	
4. Surgical removal is the mainstay for treatment.	CT shows a left adrenal adenoma measuring 2.5 cm.	
5. Medical management appropriate. Surgery is not required.	BP controlled by spironolactone. Patient is reluctant for surgery.	

Vignette 6

A 38-year-old man presents with persistent hypertension, hypokalemia, and metabolic alkalosis. See Table 13 and answer.

**Table 13:** Vignette 6 ACE: angiotensin-converting enzyme

Hypothesis	New information	Answer
1. He is suffering from secondary hyperaldosteronism.	On examination, generalized edema with ascites was present.	
2. Patient's hypertension is due to secondary hyperaldosteronism.	Laboratory shows high plasma renin activity with high aldosterone. Normal aldosterone-to-renin ratio.	
3. ACE inhibitors can be given for the management of hypertension.	Patient has a history of bilateral renal artery stenosis.	
4. Mineralocorticoid receptor antagonist for long-term management.	He has chronic kidney disease with hyperkalemia.	
5. Spironolactone treatment to be administered.	Patient has hepatic cirrhosis with ascites.	

Appendix C

Individual Readiness Assurance Test (IRAT) and Team Readiness Assurance Test (TRAT) on Adrenal Disorders

Total marks=1×10=10 marks

1. The most common cause of Cushing syndrome is:

(a) Adrenal adenoma. (b) Exogenous corticosteroid therapy. (c) Ectopic ACTH secretion. (d) Pituitary adenoma.

2. What will be the electrolyte pattern in primary adrenal insufficiency?

(a) Hyponatremia hyperkalemia. (b) Hypernatremia hypokalemia. (c) Hypernatremia hyperkalemia. (d) Hyponatremia hypokalemia.

3. A 45-year-old man with newly diagnosed hypertension is found to have low potassium levels. His labs reveal hypokalemia and metabolic alkalosis. Further testing shows a high plasma aldosterone-to-renin ratio. Which imaging study would be most appropriate next?

(a) MRI of the brain. (b) CT scan of the adrenal glands. (c) Ultrasound of the kidneys. (d) Chest X-ray.

4. A 30-year-old woman presents with fatigue, weight loss, nausea, and craving for salty foods. On examination, she has low blood pressure and generalized skin hyperpigmentation. Labs show hyponatremia and hyperkalemia. What is the most likely diagnosis?

(a) Conn syndrome. (b) Primary adrenal insufficiency (Addison's disease). (c) Cushing syndrome. (d) Secondary adrenal insufficiency.

5. In Addison's crisis, the immediate treatment is:

(a) Dexamethasone only. (b) IV fluids only. (c) IV hydrocortisone and IV fluids. (d) Spironolactone.

6. A 45-year-old woman diagnosed with Cushing's syndrome presents with proximal muscle weakness and complaints of back pain. Which of the following best explains the presence of both osteoporosis and muscle weakness in this condition?

(a) Excess cortisol increases osteoblast activity and enhances muscle growth. (b) Cortisol causes protein breakdown and osteoclastic bone resorption. (c) Cortisol increases calcium deposition in bones and hypokalemia. (d) Cortisol inhibits vitamin D absorption from the gut and protein synthesis.

7. A 35-year-old woman presents with central obesity, thin skin, purple striae, and irregular periods. Laboratory test shows elevated cortisol and elevated ACTH, and the high-dose dexamethasone suppression test shows the suppression of ACTH secretion. What is the likely diagnosis?

(a) Adrenal adenoma. (b) Pituitary adenoma. (c) Ectopic ACTH production. (d) Exogenous steroid use.

8. A 30-year-old woman presents with central obesity, a rounded face, and purple striae. She has hypertension and diabetes. She reported irregular periods, ache, and proximal muscle weakness. MRI confirms a 5 mm pituitary tumour. The initial best treatment of choice is:

(a) Transsphenoidal surgical removal of the pituitary tumour. (b) Bilateral adrenalectomy. (c) Radiotherapy. (d) Medical therapy with ketoconazole.

9. In Conn's syndrome, spironolactone improves symptoms because it:

(a) Blocks sodium channels. (b) Antagonizes the aldosterone receptor. (c) Inhibits renin. (d) Inhibits cortisol synthesis.

10. A 55-year-old male with long-standing, resistant hypertension undergoes evaluation. His lab results show hypokalemia, and his aldosterone-to-renin ratio (ARR) is elevated. Based on these findings, his physician confidently proceeds with a bilateral adrenalectomy. Evaluate the justification for the physician's decision. What is the most critical flaw in this approach?

(a) The physician failed to perform a low-dose dexamethasone suppression test to confirm hyperaldosteronism. (b) The physician did not perform adrenal venous sampling (AVS) to confirm unilateral aldosterone overproduction. (c) Bilateral adrenalectomy is never a suitable treatment for Conn's syndrome. (d) The physician did not first attempt medical management with a mineralocorticoid receptor antagonist like spironolactone.

Appendix D

Application Exercise

Total marks=3×5=15 marks

Application Exercise 1 (5 Marks)

Clinical Scenario

A 30-year-old woman presents with progressive weight gain, moon face, hypertension, irregular menstrual cycles, and proximal weakness. On examination, she has central obesity, thin extremities, purple abdominal striae, and hypertension. Laboratory findings revealed the following: Fasting blood glucose is elevated, plasma cortisol level is elevated and fails to suppress after a low-dose dexamethasone suppression test, and plasma ACTH is also high.

Q. What is the most likely diagnosis? Explain the physiological basis of her symptoms. State the investigations and treatment of the patient.

Application Exercise 2 (5 Marks)

Clinical Scenario

A 40-year-old man presents with persistent hypertension despite lifestyle modification. He complains of muscle weakness and occasional cramps. On examination, his BP was 170/100 mmHg, and no peripheral edema was noted. Laboratory findings show the following: serum sodium: elevated; serum potassium: low; plasma renin activity: suppressed; and plasma aldosterone: elevated.

Q. What is the most likely diagnosis? Explain the physiological basis of her symptoms. State the investigations and treatment of the patient.

Application Exercise 3 (5 Marks)

Clinical Scenario

A 28-year-old woman presents with progressive fatigue, weight loss, dizziness on standing, and generalized weakness for 6 months. On examination, her BP was 90/60 mmHg, and hyperpigmentation over the knuckles and buccal mucosa was noted. Laboratory findings show the following: serum sodium: low; serum potassium: high; plasma cortisol: elevated; and plasma ACTH: elevated.

Q. What is the most likely diagnosis? Explain the physiological basis of her symptoms. State the investigations and treatment of the patient.

Appendix E

Team-Based Learning (TBL) Session Feedback Questionnaire for Students

Instructions: Please provide your honest feedback on the TBL session. Your responses will help improve future sessions.

Part A: Likert Scale Questions in Table 14

Rate the following statements on a scale of 1 to 5: 1=strongly disagree, 2=disagree, 3=neutral, 4=agree, 5=strongly agree.

**Table 14:** Feedback questionnaire IRAT: Individual Readiness Assurance Test; TRAT: Team Readiness Assurance Test; TBL: team-based learning; CTAT: Critical Thinking Assessment Test; SCT: Script Concordance Test

Questions	Score
1. The IRAT was effective in assessing my preparation.	
2. The TRAT enhanced my understanding of the concepts.	
3. Working in teams improved my participation, collaboration, and willingness to share ideas.	
4. The TBL session encouraged me to analyze clinical information rather than just memorizing facts.	
5. The questions of CTAT were challenging to understand and answer.	
6. The TBL session improved my critical thinking skills.	
7. The clinical cases helped me to apply physiological concepts to real-world situations.	
8. The SCT enhanced my clinical reasoning and decision-making ability.	
9. The TBL session helped me to integrate knowledge from multiple domains while solving the cases.	
10. I prefer the TBL session over didactic lecture for in-depth understanding of the subject and improvement of interpersonal skills.	

Part B: Open-Ended Questions

1. What did you like most about the TBL session?

2. What challenges did you face during the TBL session?

3. How can the pre-session material or format be improved?

4. Were the group dynamics effective? If not, what could be done to improve them?

5. Do you have any additional suggestions for improving future TBL sessions?
